# Effect of Sophocarpine on the Pharmacokinetics of Umbralisib in Rat Plasma Using a Novel UPLC-MS/MS Method

**DOI:** 10.3389/fphar.2022.749095

**Published:** 2022-01-20

**Authors:** Qinghua Weng, Xia Lan, Yingjie Wang, Chen Fan, Ren-ai Xu, Pengzhao Zhang

**Affiliations:** ^1^ The Third Affiliated Hospital of Shanghai University (Wenzhou People’s Hospital), Wenzhou, China; ^2^ Chongqing University Cancer Hospital, Chongqing, China; ^3^ School of Basic Medical Sciences, Henan University of Science and Technology, Luoyang, China; ^4^ The First Affiliated Hospital of Wenzhou Medical University, Wenzhou, China; ^5^ The People’s Hospital of Lishui, Lishui, China

**Keywords:** umbralisib, phosphatidylinositol 3-kinase delta, casein kinase 1 epsilon, pharmacokinetic, UPLC-MS/MS, rat

## Abstract

Umbralisib is a dual inhibitor of phosphatidylinositol 3-kinase delta (PI3Kδ) and casein kinase 1 epsilon (CK1ε) for treating marginal zone lymphoma (MZL) and follicular lymphoma (FL). This study aimed to develop a fast and stable ultra performance liquid chromatography tandem mass spectrometry (UPLC-MS/MS) method for quantitative analysis of umbralisib in rat plasma and its application for evaluating the effect of sophocarpine on the pharmacokinetics of umbralisib. A direct protein preparation with acetonitrile was used to deal with rat plasma. Umbralisib and duvelisib (internal standard, IS) were isolated on a Waters Acquity UPLC BEH C18 column with mobile phase consisted of acetonitrile and 0.1% formic acid in water. The linear range was from 0.5 to 1,000 ng/ml. Both of the precision (RSD%) and accuracy (RE%) were less than 15% in a permissible range. The mean recovery and matrix effect of umbralisib were 86.3–96.2% and 97.8–112.0%, respectively. When umbralisib was combined with sophocarpine, AUC_0→∞_ of umbralisib was significantly reduced to 2462.799 ± 535.736 ng/ml•h from 5416.665 ± 1,451.846 ng/ml•h, and C_max_ also was markedly diminished. Moreover, CLz/F was increased more than two times. This developed, optimized and technical UPLC-MS/MS method was extremely suitable for detecting the concentrations of umbralisib in rat plasma after an oral administration, and sophocarpine significantly changed the pharmacokinetics of umbralisib in rats. This obvious pharmacokinetic changes indicates that there seems to exist herb-drug interaction between sophocarpine and umbralisib.

## Introduction

Marginal zone lymphoma (MZL) and follicular lymphoma (FL) are two common subtypes of non-Hodgkin lymphoma, which appears a relatively high prevalence with 500,000 new cases and mortality with 259,793 new deaths in 185 countries according to the global cancer statistics 2020 (Sung et al., 2021). It brings not only an economic burden for the family, but also a big challenge for the healthcare system and society (Monga et al., 2019). Therefore, more effective and novel treatments for MZL and FL were needed and developed.

**FIGURE 1:**
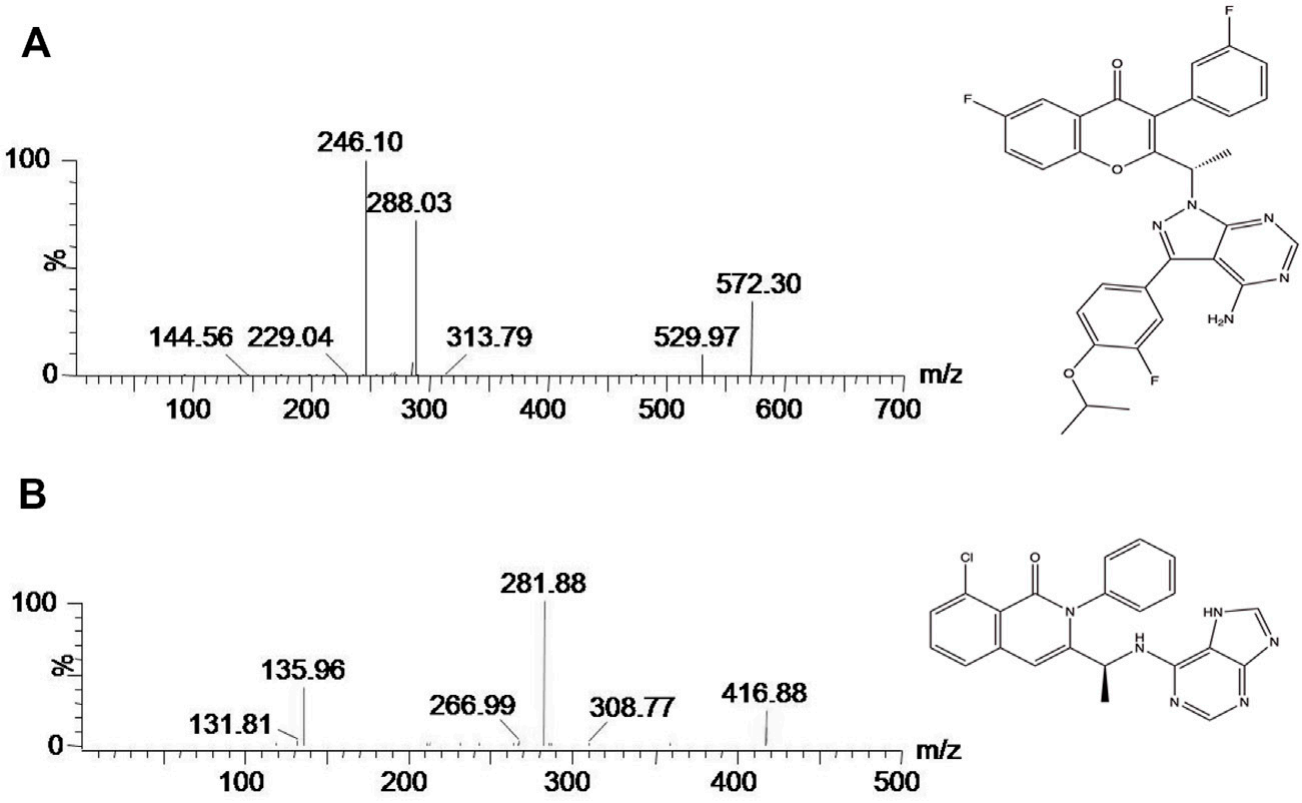
Mass spectra of umbralisib **(A)** and duvesilib [IS, **(B)**] in this study.

Phosphatidylinositol 3-kinase delta (PI3Kδ) inhibitors are proved to be promising drugs for treating lymphoma (Fan et al., 2020; Coleman et al., 2021), and casein kinase 1 epsilon (CK1ε) is also a potent target for the therapy of lymphoma. Nowadays, umbralisib (Figure 1A) has become the first approved, oral and dual inhibitor of PI3Kδ and CK1ε in the treatment for refractory or recurrent MZL and FL (Dhillon and Keam, 2021; Fowler et al., 2021). Following administration of a single dose of umbralisib in healthy subjects, the median time to reach peak plasma concentration (C_max_) of umbralisib is ≈4 h (TGTherapeutics, 2021. https://www.tgtherapeutics.com/. Accessed 23 Feb 2021). As seen from a previous study, umbralisib plasma concentrations in humans were found not to display any clinically significant differences in human demography (age, sex, race and body weight) and some clinical manifestations (mild hepatic/renal impairment) (FDA, 2021. Available: https://www.tgtherapeutics.com/prescribing-information/uspi-ukon.pdf [Accessed 2021]). Interestingly, umbralisib is mainly metabolized by three isoforms of CYP450 enzymes *in vitro*, including CYP2C9, CYP3A4, and CYP1A2 (TG Therapeutics. UKONIQTM (umbralisib): United States prescribing information. 2021. https://www.tgtherapeutics.com/. Accessed 23 Feb 2021). Thus, we couldn’t ignore its herb-drug interactions in combination with other drugs. Few studies of coadminstration with other drugs metabolized by these three CYP450 enzymes were reported due to its recent approval, so it is important to explore its potential herb-drug interactions with other drugs.

As reported, Compound kushen injection combination with other chemotherapies improved the efficiency and reduced the adverse effect rate in the treatment of lymphoma. In addition, Compound kushen injection is commonly used for reliefing cancer pain according to its package insert. A biologically active chemical alkaloid, sophocarpine, is separated from Compound kushen injection (Zhang et al., 2018)*.* As reported before, sophocarpine might be a potential broad-spectrum anticancer agent (Zhang et al., 2016; Huang et al., 2019; Wang et al., 2019; Yang et al., 2021). What’s more, sophocarpine also inhibited CYP3A4 noncompetitively and CYP2C9 competitively in human liver microsomes (Zhang et al., 2019). Therefore, in this research, we decided to investigate the pharmacokinetic drug interactions between umbralisib and sophocarpine based on the possibility of co-administrations and interactions theoretically.

To the best of our knowledge, no quantitative analytical method was developed for the measurement of umbralisib in biological fluids from pharmacokinetics and herb-drug interactions study. In this experiment, we tried to establish a novel, stable and sensitive ultra performance liquid chromatography tandem mass spectrometry (UPLC-MS/MS) assay in the application for quantitative analysis of the concentrations of umbralisib in rat plasma, and it was successfully applied in the investigation of the effect of sophocarpine on the pharmacokinetics of umbralisib in rats.

## Materials and Methods

### Chemicals and Materials

All chemicals and reagents were showed at Table 1.

**TABLE 1:** List of chemicals and reagents.

Name	Source
Duvelisib^a^/Umbralisib^a^	Shanghai Chuangsai Technology Co., Ltd. (Shanghai, China)
Methanol^b^/Acetonitrile^b^	Merck Company (Germany)
Formic acid^c^	Shanghai Aladdin Biochemical Technology Co., Ltd. (China)
Ultrapure water	Milli-Q Water Purification System (United States)

a The purity of Chemicals is >98%.

b All reagents are HPLC, grade.

c All reagents are AR, grade.

### Animal Experiments

A total of 12 male Sprague-Dawley (SD) rats were obtained from the Laboratory Animal Center of Wenzhou Medical University (Zhejiang, China). Experimental procedures were conducted in accordance with the National Institute of Health (NIH) guidelines for the welfare and use of animals, and were finally approved by the Institutional Ethics Committee of Wenzhou Medical University. SD rats weighted 200 ± 20 g were adaptively kept in a controlled feeding room (standard temperature 25–28°C, humidity 50–60% and 12 h light/12 h dark) for seven consecutive days. All rats acquired water and food without restriction.

All rats were equally divided into two groups, including the control group (umbralisib) and the intervention group (sophocarpine + umbralisib). A 12 h fasting for rats was needed before animal experiments, but unlimited water was allowed. The intervention group was orally administrated with 20 mg/kg sophocarpine dissolving in carboxymethyl cellulose sodium (CMC-Na) for 7 days, while the control group was orally administrated with equal volume CMC-Na solution. 80 mg/kg umbralisib was orally given to all SD rats after 0.5 h of the last oral sophocarpine/CMC-Na solution. The blood samples derived from the caudal vein were collected to a 1.5 ml of heparin-covering Eppendorf tube at different time points of 0, 0.333, 0.667, 1, 1.5, 2, 3, 4, 6, 8, 12, 24, 48, and 72 h. After centrifuging (4,000 × *g*, 25°C, 8 min), the supernatants were harvested and stored at −80°C for further quantitative analysis using a developed UPLC-MS/MS method.

### Instrumentations and Analytical Conditions

A complete UPLC-MS/MS system was composed of Waters ACQUITY UPLC I-Class system (Milford, MA, United States) and Waters Xevo TQ-S triple quadrupole tandem mass spectrometer. To collect and further process the experimental data, the system-provided Quanlynx programme and Masslynx 4.1 software (Milford, MA, United States) were needed.

The separation was performed on a Waters ACQUITY UPLC I-Class system via an Acquity UPLC BEH C18 column (2.1 mm × 50 mm, 1.7 μm) with the temperature of 40°C. All samples were temporarily stored at an autosampler (10°C), and 2.0 µl of injection volume for each specimen was pushed into UPLC-MS/MS system for further analysis. A gradient elution program composed of solvent A (acetonitrile) and solvent B (0.1% formic acid in water) at a total flow rate of 0.40 ml/min was applied as follows: first, 10% A kept for half a minute; then, the proportion of A linearly increased to 90% in half a minute, maintained this proportion of A for 0.6 min; finally, the proportion of A linearly decreased to 10% in the next 0.1 min, and maintained for 0.3 min. Total consumption of time was 2.0 min. Time remaining was used for the equilibrium.

A Xevo TQ-S triple quadrupole tandem mass spectrometer equipped with an electro-spray ionization (ESI) source (Milford, MA, United States) was used to determine the concentrations of the target and IS. All quantitative analysis was based on a selective reaction monitoring (SRM) mode and positive ion mode. Ion transitions of umbralisib and IS were *m/z* 572.30 → 246.10 and *m/z* 416.88 → 281.88, respectively.

The flow rate of collision gas, cone gas and desolvation gas filled with high purity of nitrogen was 0.15 ml/min, 200 L/h and 1000 L/h, respectively. The optimized desolvation temperature reached 600°C. Collision energy of umbralisib and IS was separately 30 and 20 eV, and cone voltage was separately 15 and 20 V. Capillary voltage was finally optimized to be 2.0 kV.

### Standard Solutions, Calibration Curves and Quality Control (QC) Samples

Stock solutions of umbralisib and IS were dissolved into the same final concentration of 1.00 mg/ml using methanol. Working solution of umbralisib was further dissolved in methanol via the corresponding stock solutions to form a series of concentrations ranged from 5 to 10,000 ng/ml. Working solution of IS was achieved at an ultimate concentration of 200 ng/ml in the same method. Calibration curve was established at a series of concentrations using working solution spiked with blank rat plasma. Quality control samples (QCs) with four different final concentrations of 0.5, 1, 80 and 800 ng/ml were also served in the similar method. An ultra deep-freeze equipment was used as temporary storage for all the stock and working solutions.

### Sample Preparation

Plasma preparation adopted a simple method of protein precipitation with acetonitrile. Firstly, 20 µl of IS working solution was added and mixed with five times volume of plasma specimens in an EP centrifuge tube (1.5 ml). Then, another addition of 300 µl acetonitrile was used as a protein precipitation solution to remove protein. Subsequently, sufficient vortexing (1 min) and centrifuging (13,000 g, 4°C, 10 min) were needed. Finally, a 100 µl aliquot of clear supernatant was transferred into autosampler vials for subsequent detection.

### Method Validation

In this study, we strictly and exactly adhered to the principles of FDA based on the validation of bioanalytical assay (Xu et al., 2019; Tang et al., 2020). Generally, a complete methodology validation is selected to cover the calibration curve, selectivity, lower limit of quantification (LLOQ), recovery, matrix effect, precision and accuracy, and stability.

To validate the selectivity of the method and check the absence of interferences at the nearby retention times of umbralisib and IS, we investigated and assessed three kinds of analytic samples, including blank rat plasma, standard solution (at the concentration of LLOQ) and plasma samples derived from rats with oral administration of umbralisib.

Sensitivity was systematically assessed by the value of LLOQ, which was identified as the lowest quantitative point and evaluated as 10 times of signal-to-noise ratio (S/N). Calibration curves were investigated in a weighted (1/*x* ^2^) least square regression model. In this work, calibration curves represented the ratio of peak area of the analyte to peak area of IS against the nominal concentrations of the analyte.

Six replicates plasma specimens in each level were finally detected and applied in the estimation of precision, accuracy, recovery and matrix effect. The evaluation of precision and accuracy was performed using QC samples at four different concentrations including 0.5, 1, 80, 800 ng/ml in three continuous days. Recovery was recognized as the peak area of umbralisib before and after plasma preparation. Matrix effect was investigated by comparative study between the peak area of the spike-after-extraction samples and the corresponding results of the pure solution.

Stability (short-term and long-term) evaluation was examined respectively at ambient conditions temperature for 2 h and −80°C for 3 weeks. In addition, the stability after 4 h of preparation was tested in an autosampler at 10°C. Moreover, three complete freeze/thaw stability (−80°C to room temperature) was also studied. Stability of umbralisib with five replicates was studied at three different levels containing 1, 80 and 800 ng/ml.

### Statistical Analysis

Graphpad prism 8.0 was used for drawing the diagram of mean plasma concentration-time curves. A non-compartmental method to calculate the pharmacokinetic parameters was employed through Drug and Statistics (DAS) 3.0 software (Mathematical Pharmacology Professional Committee of China, Shanghai, China). One-way ANOVA analysis was applied to compare the pharmacokinetic parameters between different groups (*p* < 0.05) using SPSS (version 17.0; SPSS Inc., Chicago, IL, United States). *p* < 0.05 is statistically significant.

## Results and Discussion

### Method Development and Optimization

To get better analytic results, we optimized the MS parameters and acquired better settings using umbralisib and IS. Figure 1 was mass spectrum of umbralisib and IS. The parent ion of umbralisib was *m/z* 572.30, and the corresponding ion of IS was 416.88. The most abundant fragment ion at *m/z* 246.10 and 281.88 separately belonged to umbralisib and IS, which were chosen as the product ions for the bioanalytical method.

The selection of mobile phase was an important procedure of bioanalytical method. Gradually, acetonitrile or methanol for the organic phase was investigated, and water with acetic acid, or formic acid or ammonium acetate for water phase was evaluated to acquire the satisfied sensitivity and symmetric peaks. In this investigation of the developed method, we finally used eight combinations to evaluate the sensitivity, including four kinds of water phase (water, 0.1% acetic acid solution, 0.1% formic acid solution and 1 mM ammonium acetate solution) and two kinds of organic phase (acetonitrile and methanol). Finally, 0.1% formic acid solution as water phase and acetonitrile as mobile phase were adopted and acquired the best sensitivity and acceptable symmetric peaks.

Plasma is filled with large amount of endogenous substances and protein is one kind of complicated matrix effect. Plasma specimens must be removed the interferences as much as possible before UPLC-MS/MS analysis. Compared with liquid-liquid extraction method, direct protein precipitation has the unique superiority with simpler process and less time consumption. One-step protein precipitation was the more effective method to reduce the endogenous interferences and to improve the recovery in plasma samples (Wei et al., 2020; Zhu et al., 2020). In this study, we used acetonitrile to directly process rat plasma samples and got a good spectrum and an acceptable recovery.

### Method Validation

#### Selectivity

**FIGURE 2:**
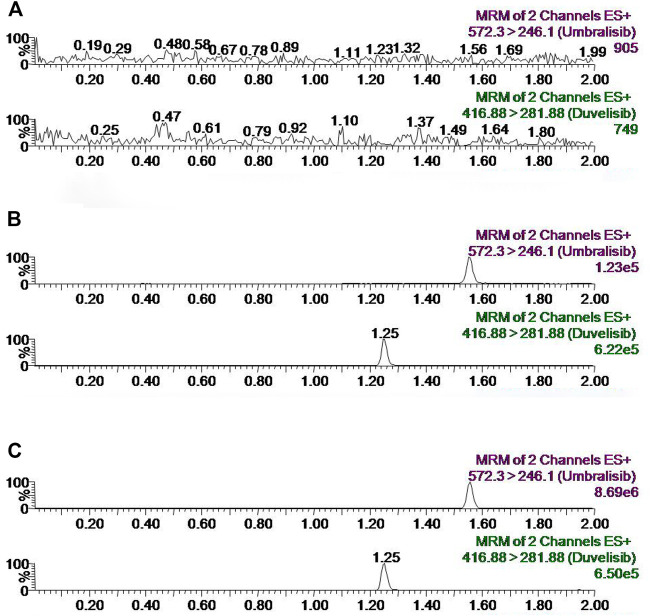
Representative chromatograms of umbralisib and IS in rat plasma: **(A)** blank plasma; **(B)** blank plasma spiked with LLOQ concentration of standard solutions; **(C)** sample obtained from a rat at 0.333 h after oral administration of 80 mg/kg umbralisib and dealed with preparation with IS.

Figure 2 represented chromatograms of umbralisib and IS in different plasma samples, including blank plasma, blank plasma with standard solutions and plasma specimens with standard preparations after gavage administration of the analyte. Retention times of umbralisib and IS separately achieved at 1.55 and 1.25 min. No potential interfering substances were significantly observed according to the chromatograms. These results suggested that the method had a good selectivity in the determination of umbralisib and IS in rat plasma.

#### Calibration Curve and LLOQ

The calibration curve consisted of umbralisib with eight different concentrations ranged from 0.5 to 1,000 ng/ml. The calibration curve was Y = 5.95626 × X + 12.211 (y is described as the value of the peak area ratio of umbralisib against IS and x is expressed as the plasma levels of umbralisib), and the correlation coefficient (*r* ^2^) reached 0.996,676, which exhibited good linearity. A concentration of 0.5 ng/ml was used for the evaluation of LLOQ, which was validated in an acceptable limit.

#### Precision and Accuracy

The precision was expressed as relative standard deviation (RSD%), and the accuracy was represented as relative error (RE%). The estimation of accuracy and precision were divided into two kinds, including inter-day and intra-day, which were showed in Table 2. From the results, we conducted that the highest values of intra-day precision (12.2%) and inter-day precision (14.9%) were appeared at the lowest concentration of umbralisib, and the corresponding values of intra-day accuracy (13.6%) and inter-day accuracy (10.0%) were showed at a concentration of 1 ng/ml umbralisib. Together with these results suggested the precision and accuracy were within the permissible limit of FDA standard guidelines.

**TABLE 2:** The precision and accuracy of umbralisib in rat plasma (*n* = 6).

Analyte	Concentration (ng/ml)	Intra-day		Inter-day	
		RSD%	RE%	RSD%	RE%
umbralisib	0.5	12.2	6.1	14.9	1.8
	1	10.5	13.6	11.9	10.0
	80	6.1	−0.8	5.7	−0.4
	800	2.4	1.0	3.5	−1.0

#### Recovery and Matrix Effect

The mean recovery and matrix effect of umbralisib were 86.3–96.2% (Table 3) and 97.8–112.0% (Table 3), respectively, which were within the bounds of FDA guidelines. These results showed that an excellent recovery and no significant matrix effects were found in the methodology of UPLC-MS/MS establishment.

**TABLE 3:** Recovery and matrix effect of umbralisib in rat plasma (*n* = 6).

Analyte	Concentration added (ng/ml)	Recovery (%)		Matrix effect (%)	
		Mean ± SD	RSD (%)	Mean ± SD	RSD (%)
umbralisib	1	86.3 ± 10.74	12.4	112.0 ± 10.87	9.7
	80	92.1 ± 2.75	3.0	99.5 ± 6.06	6.1
	800	96.2 ± 7.13	7.4	97.8 ± 7.84	8.0

#### Stability

The results of the stability of umbralisib under different storage and preparation conditions in rat plasma were listed in Table 4. It was suggested that the stability of umbralisib was especially stable. Among them, RSD% ranged 1.6–14.2%, and RE% was from −4.8 to 11.4%.

**TABLE 4:** Stability results of umbralisib in plasma under different conditions (*n* = 6).

Analyte	Added (ng/ml)	Room temperature, 2 h		Autosampler 10°C, 4 h		Three freeze-thaw		−80 °C, 3 weeks	
		RSD (%)	RE (%)	RSD (%)	RE (%)	RSD (%)	RE (%)	RSD (%)	RE (%)
umbralisib	1	7.6	14.3	11.1	14.0	11.4	13.9	10.6	11.6
	80	3.2	−2.6	5.0	−2.8	2.3	−4.8	4.0	−1.2
	800	1.9	1.8	3.4	1.7	1.6	0.5	2.2	−0.6

#### Animal Study

**FIGURE 3:**
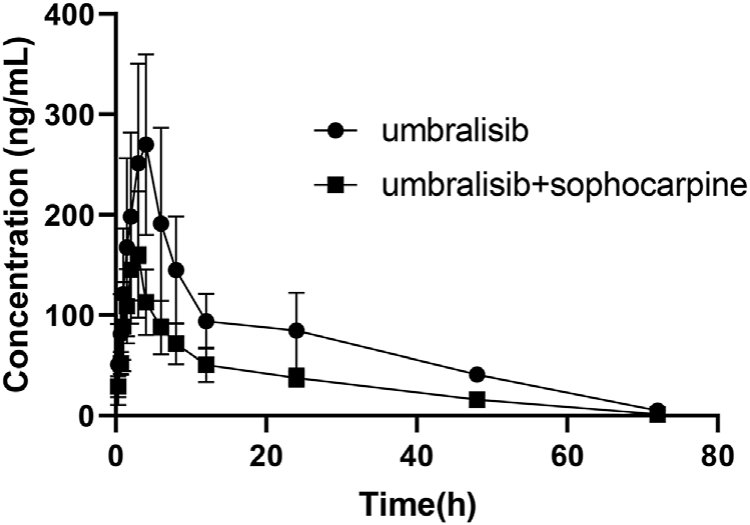
Mean plasma concentration-time curves of umbralisib in rats after oral administration of umbralisib with or without sophocarpine (*n* = 6).

Table 5 presented the main pharmacokinetic results acquired from a non-compartment model analysis in the combination with or without sophocarpine. Figure 3 displayed the average plasma concentration-time curves of umbralisib or combination with sophocarpine in rats. As seen from the results of pharmacokinetic parameters, it was quickly absorbed that the time of maximum concentration (T_max_) was 3.667 ± 0.516 h, which was similar with the reported result in human (Burris et al., 2018). And the corresponding maximum plasma concentration (C_max_) reached 283.803 ± 84.714 ng/ml. We also could recognize umbralisib as a long-acting preparation with half-life (t_1/2_) reached 16.864 ± 5.982 h.

**TABLE 5:** The main pharmacokinetic parameters of unbralisib with or without sophocarpine in rats (*n* = 6, Mean ± SD).

Parameters	Umbralisib	Umbralisib + sophocarpine
AUC_0→t_ (ng/mL•h)	5114.90 ± 1,515.34	2413.34 ± 537.20^a^
AUC_0→∞_ (ng/mL•h)	5416.67 ± 1,451.85	2462.80 ± 535.74^a^
MRT_0→t_ (h)	20.92 ± 1.22	18.65 ± 2.17
MRT_0→∞_ (h)	22.06 ± 1.87	19.00 ± 2.04
t_1/2_ (h)	16.86 ± 5.98	12.06 ± 3.93
T_max_ (h)	3.67 ± 0.52	2.83 ± 0.41^a^
Vz/F (L/kg)	382.19 ± 158.08	587.06 ± 211.55^a^
CLz/F (L/h/kg)	15.49 ± 3.34	33.97 ± 8.29^a^
C_max_ (ng/ml)	283.80 ± 84.71	166.01 ± 56.39^a^

a Represents *p* < 0.05.

After combination with sophocarpine, significant alterations were found in the pharmacokinetic parameters. Among them, area under the curve for plasma concentration from zero to last measurable plasma sample time (AUC_0-t_) and the area under the curve for plasma concentration from zero to infinity (AUC_0-∞_) were separately significantly reduced to 2413.341 ± 537.196 ng/ml•h and 2462.799 ± 535.736 ng/ml•h, C_max_ was dramatically reduced to 166.011 ± 56.387 ng/ml, and T_max_ was obviously reduced by nearly 1.0 h. It suggested that sophocarpine had significantly increased the metabolism of umbralisib in rats. Therefore, the concurrent use of umbralisib with sophocarpine should be treated with extreme caution. If their combined use is unavoidable, our data suggested that dose increase should be taken. Otherwise, the patient might suffer from the failure of the treatment caused by decreased umbralisib plasma levels. However, it was reported that sophocarpine had the potential inhibitory effects on CYP3A4 and CYP2C9 *in vitro* using human liver microsomes (Zhang et al., 2019), which was not consistent with our results in rats. The limitation of our research lies in the small number of rats used in the experiment. These results may aid in investigating the role of sophocarpine on umbralisib metabolism, and the potential of herb-drug interactions in humans in future studies should be done to evaluate the significance of this interaction.

## Conclusion

In conclusion, we firstly established a reliable, quick and robust UPLC-MS/MS method to quantify the concentrations of a novel anticarcinoma drug umbralisib in rat plasma. Meanwhile, UPLC-MS/MS method was successfully applied in the pharmacokinetics of umbralisib with or without a potent anti-tumor agent sophocarpine in rats. What’s more, we found sophocarpine had significantly increased the metabolism of umbralisib in rats. It indicates that it is essential for medical personnel to pay more attention to herb-drug interactions in further clinical use.

## Data Availability

The raw data supporting the conclusion of this article will be made available by the authors, without undue reservation.
